# A design of forceps-type coincidence radiation detector for intraoperative LN diagnosis: clinical impact estimated from LNs data of 20 esophageal cancer patients

**DOI:** 10.1007/s12149-021-01701-9

**Published:** 2021-11-29

**Authors:** Miwako Takahashi, Shuntaro Yoshimura, Sodai Takyu, Susumu Aikou, Yasuhiro Okumura, Koichi Yagi, Masashi Fukayama, Toshimitsu Momose, Yasuyuki Seto, Taiga Yamaya

**Affiliations:** 1 Department of Advanced Nuclear Medicine Sciences, Institute for Quantum Medical Science, National Institutes for Quantum and Radiological Science and Technology, 4-9-1 Anagawa, Inage-ku, Chiba, 263-8555 Japan; 2 Department of Gastrointestinal Surgery, Graduate School of Medicine, The University of Tokyo, Tokyo, Japan; 3 Department of Pathology, Graduate School of Medicine, The University of Tokyo, Tokyo, Japan; 4 Department of Nuclear Medicine, Graduate School of Medicine, The University of Tokyo, Tokyo, Japan

**Keywords:** 18F-FDG, PET, Esophageal cancer, Lymph node metastasis, Forceps

## Abstract

**Purpose:**

To reduce postoperative complications, intraoperative lymph node (LN) diagnosis with ^18^F-fluoro-2-deoxy-D-glucose (FDG) is expected to optimize the extent of LN dissection, leading to less invasive surgery. However, such a diagnostic device has not yet been realized. We proposed the concept of coincidence detection wherein a pair of scintillation crystals formed the head of the forceps. To estimate the clinical impact of this detector, we determined the cut-off value using FDG as a marker for intraoperative LN diagnosis in patients with esophageal cancer, the specifications needed for the detector, and its feasibility using numerical simulation.

**Methods:**

We investigated the dataset including pathological diagnosis and radioactivity of 1073 LNs resected from 20 patients who underwent FDG-positron emission tomography followed by surgery for esophageal cancer on the same day. The specifications for the detector were determined assuming that it should measure 100 counts (less than 10% statistical error) or more within the intraoperative measurement time of 30 s. The detector sensitivity was estimated using GEANT4 simulation and the expected diagnostic ability was calculated.

**Results:**

The cut-off value was 620 Bq for intraoperative LN diagnosis. The simulation study showed that the detector had a radiation detection sensitivity of 0.96%, which was better than the estimated specification needed for the detector. Among the 1035 non-metastatic LNs, 815 were below the cut-off value.

**Conclusion:**

The forceps-type coincidence detector can provide sufficient sensitivity for intraoperative LN diagnosis. Approximately 80% of the prophylactic LN dissections in esophageal cancer can be avoided using this detector.

## Introduction

Surgery is the mainstay of cancer treatment, since almost all tumor cells can be resected over several hours. According to the annual reports on gastrointestinal surgical outcomes in the Japanese National Clinical Database, postoperative mortality rates have been decreasing in the past 7 years. However, the complication rates are gradually increasing [1]. The report showed that the complication rate was 22.2% in esophageal surgery, which was the second highest rate following that in pancreatic surgery (22.5%) despite the progress in surgical procedures such as thoracoscopic, laparoscopic, and robot-assisted surgeries [2]. The high complication rate is related to the large extent of lymph node (LN) dissection [3–6]. In particular, pulmonary inflammation is one of the lethal complications, mainly due to swallowing dysfunction caused by muscle and nerve impairments in the area of the cervical LNs and the recurrent laryngeal nerve LN stations [4, 5, 7]. Currently, the standard surgery for esophageal cancer is associated with three-field or two-field dissection, since LN metastasis tends to develop even in the early stages and the location of LN metastasis varies from the cervical to the abdominal field regardless of the primary tumor location [8]. However, the number of metastatic LNs among the total resected LNs is not always high. Nishihira et al. showed that the rates of LN metastasis ranged from 0% to 27.6% [3]. If we diagnose LN during surgery, we can avoid resection of the LN station, which would lead to preservation of the vessels and the nerves included in the LN stations. Therefore, to reduce the complication rates, optimization of the extent of LN dissection based on LN diagnosis in individual patients is desired.

For diagnosis of LN during surgery, ^18^F-fluoro-2-deoxy-D-glucose (FDG) may be a marker of metastatic LN diagnosis, as preoperative FDG-positron emission tomography (FDG-PET) clearly shows a marked increase in the esophageal cancer cells and the degree of FDG uptake is correlated with the LN metastatic stage [9–11]. In our previous study [12], we performed FDG-PET in 20 patients with esophageal cancer, followed by surgical treatment on the same day. Subsequently, we measured the radioactivity of each of the 1073 dissected LNs using a well-type counter and compared the radioactivity with the pathological diagnosis of each LN. We found that the radioactivity measured by the well-type counter of each LN was significantly higher in metastatic LNs than in non-metastatic LNs and the diagnostic ability had a sensitivity of 94.7% and a specificity of 78.7%, while preoperative FDG-PET imaging provided a sensitivity of 28.6% and a specificity of 96.7%. Therefore, we believe that FDG is a potentially useful marker for the detection of metastatic LNs if we can measure the radioactivity of each LN intraoperatively.

To measure radioactivity intraoperatively, we propose the concept of a forceps-type coincidence detector, which can measure coincident pairs of annihilation photons from ^18^F using two small scintillation crystals. The tow scintillation crystals can form the head of the forceps. In this study, we determine the most efficient intraoperative cut-off value to differentiate metastatic LNs from non-metastatic LNs using the clinical data obtained in our previous study. Then, setting the materials and size of the detector, the feasibility of measuring the cut-off value within 10% statistical error is confirmed by numerical simulation. Finally, the diagnostic ability for each LN station using the cut-off value is estimated.

## Materials and methods

### Clinical data

We analyzed the clinical data of 1073 LNs obtained from 20 patients with esophageal cancer, reported previously [12]. Details of the patients and the protocol of the clinical study were described previously. Briefly, all patients fasted for at least 5 h before FDG injection, and a blood glucose level below 150 mg/dL was required at the time of FDG injection. Each patient was administered 4.5 MBq/kg (0.12 mCi/kg) of FDG approximately 3 h before the scheduled time of surgery. FDG-PET/CT scanning was started at 50 min after the injection using a PET/CT scanner (Aquiduo; Toshiba Medical System, Otawara, Japan) as a routine clinical examination. At 1 pm on the same day, the patients underwent either Ivor Lewis or McKeown esophagectomy with two-field or three-field lymphadenectomy and gastric conduit reconstruction via the posterior mediastinal route. After dissection of the LNs, we harvested all LNs from the extracted specimens and measured their radioactivity in counts per second (cps) using a well-type counter (CAPRAC-t, Capintec, Inc., Pittsburgh, PA, USA). The pathological diagnosis was made by two expert pathologists blinded to the information regarding radioactivity. The patient distribution among different p-stages was as follows: 2 patients in stage 0, 4 in IA, 2 in IB, 1 in IIA, 5 in IIB, 2 in IIIA, 3 in IIIB, and 1 in IIIC based on the seventh edition of the American Joint Committee on Cancer TNM cancer staging manual for the esophagus [13]. This study was performed in accordance with the Declaration of Helsinki and was approved by the institutional review board of the institute’s hospital, and all study participants provided informed consent.

### Determination of the cut-off value during surgery

To find the most effective cut-off value to differentiate metastatic from non-metastatic LN, the values of radioactivity were also adjusted by either the LN weight, the shortest, or the longest LN diameter. The assumed time of intraoperative LN measurement was set at 6 after the FDG injection. Decay correction was applied to the radioactivity values after the conversion from cps to Bq using a cross-calibration factor of 5.9 Bq/cps between the well-type counter (cps) and the dose calibrator for FDG (Bq).

### The specifications required for the forceps-type detector and the simulation study

The specifications required for detectors to differentiate metastatic from non-metastatic LN during surgery are to be able to measure the cut-off value under the 10% or lower statistics error, which is corresponding to 100 counts based on the Poisson distribution of radioactive decay. The ideal measurement time is within 30 s for each LN during surgery.

**Fig. 1:**
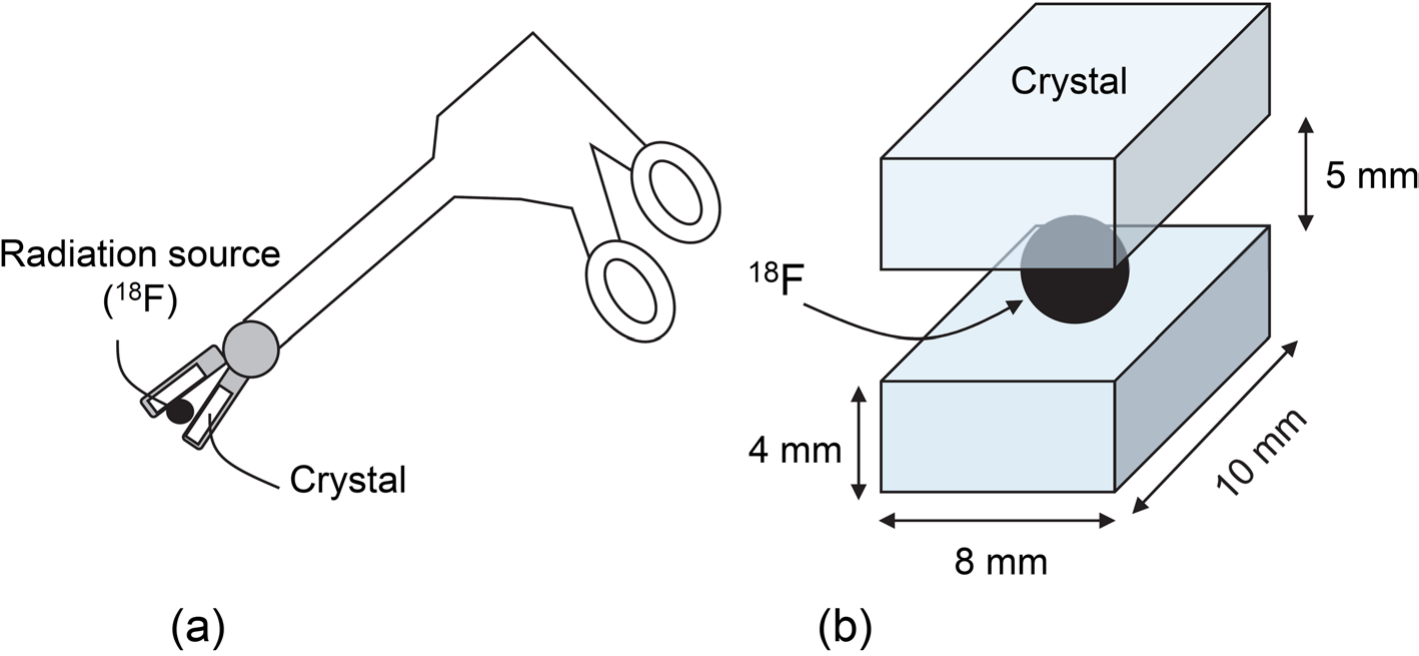
A conceptual illustration of the proposed forceps-type coincidence detector (**a**), and **b** simulated geometry of the scintillation crystals and the radiation source (^18^F)

Furthermore, to be used in laparoscopic surgery, the detector was designed to be at the maximum size of 12 mm diameter, which is inner diameter of typical trocar for laparoscopy. The detector should be composed of two scintillators for coincident measurement, therefore, Bi_4_Ge_3_O_12_ (BGO) was selected as a high-density scintillator to maximize the efficiency in the limited size, and the cross-sectional size of each BGO crystal was set at 4 mm × 8 mm for passing thorough a trocar. Then, we called this detector as a forceps-type detector because the detector can be used like a forceps (Fig. 1a).

To confirm the feasibility of the forceps-type coincidence detector to measure the most effective cut-off value, the detector efficiency was simulated using geometry and tracking (GEANT4) [14]. The configuration of the forceps-type coincidence detector was set as shown in Fig. 1a, b radiation source (^18^F) was provided in a sphere of 5-mm diameter, which was located at the center of the crystals. The coincidence time window was set to 1 ns.

Finally, diagnostic ability to diagnose LN during surgery was estimated based on the 1073 LNs data using the most effective cut-off value.

### Statistical analysis

The relationships between the radioactivity of the LNs and the weight, shortest diameter, and longest diameter of the LNs were tested using Spearman’s correlation coefficient. Statistical significance was set at p < 0.05. ROC curve analysis was performed to determine the most effective cut-off values and the area under the curve (AUC) was calculated for each measurement. Statistical analyses were performed using IBM SPSS Statistics version 26 (IBM Corp., Armonk, NY, USA).

## Results

**Fig. 2:**
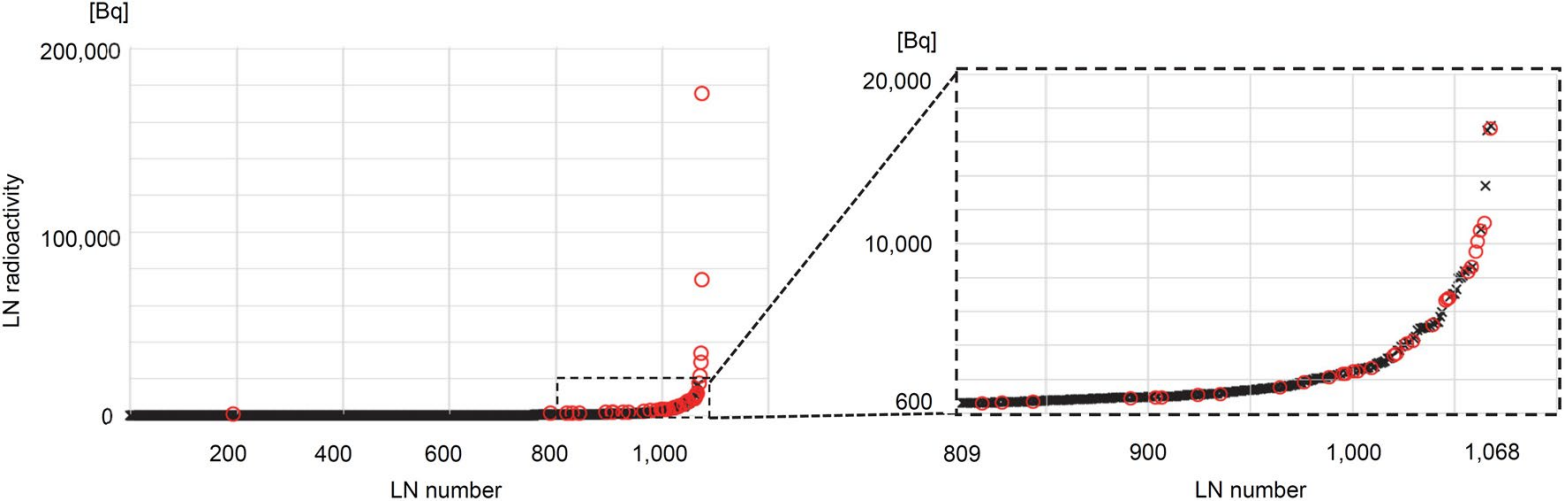
A plot of radioactivity of all lymph nodes. Lymph nodes (LNs) were numbered according to the amount of radioactivity from low to high (represented on the x-axis). The plot on the right shows an expanded scale for the range of 600 Bq–16,943 Bq (the range of 809–1068 for the LN number). Red circles indicate metastatic LNs and black crosses indicate non-metastatic LNs

The radioactivity and pathological diagnoses of all LNs are plotted in Fig. 2 The radioactivity of metastatic and non-metastatic LNs overlapped mainly in the range of 600 Bq–16,943 Bq. The plot on the right in Fig. 2 shows this range using an expanded scale.

**Fig. 3:**
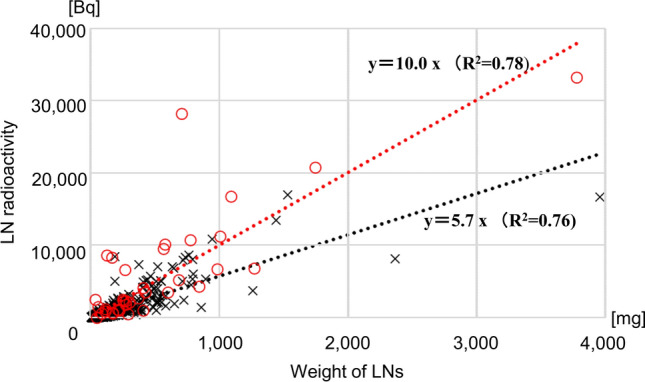
A plot of lymph node radioactivity and lymph node weight. Dashed lines indicate the first-order linear approximation equations. Significant correlations were observed between lymph node (LN) radioactivity and LN weight in metastatic LNs (*r* = 0.726, *p* < 0.001) and in non-metastatic LNs (*r = *0.870, *p* < 0.001). Red circles indicate metastatic LNs and black crosses indicate non-metastatic LNs

**Fig. 4:**
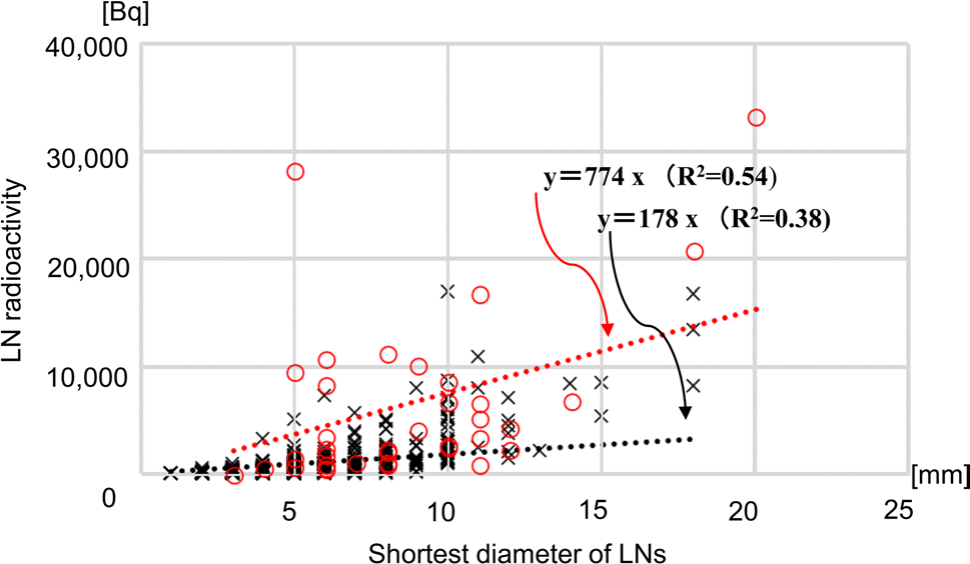
A plot of lymph node radioactivity and the shortest lymph node diameter. Lines indicate the first-order linear approximation equations. Significant correlations were observed between lymph node (LN) radioactivity and the shortest LN diameter in metastatic LNs (*r* = 0.437, *p* < 0.008) and in non-metastatic LNs (*r* = 0.739, *p* < 0.001). Red circles indicate metastatic LNs and black crosses indicate non-metastatic LNs

**Fig. 5:**
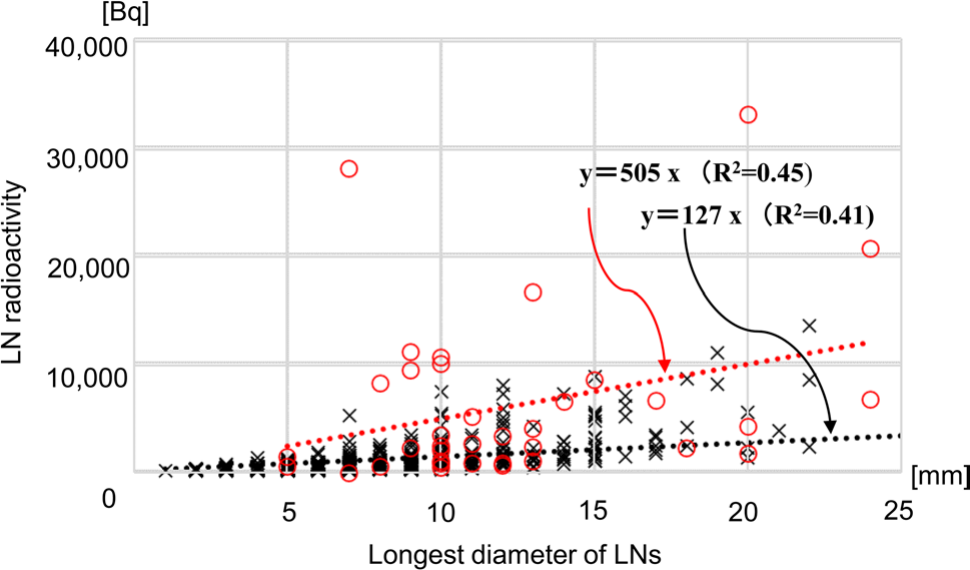
A plot of lymph node radioactivity and the longest lymph node diameter. Lines indicate the first-order linear approximation equations. No significant correlation was observed between lymph node (LN) radioactivity and the longest LN diameter in metastatic LNs (*r* = 0.287, *p* = 0.089). The correlation was significant in non-metastatic LNs (*r* = 0.730, *p* < 0.001). Red circles indicate metastatic LNs and black crosses indicate non-metastatic LNs

The relationships between the radioactivity and the weight, shortest diameter, and longest diameter of each LN are shown in Figs. 3, 4, 5. The two LNs with the highest radioactivity (LN numbers 1073 and 1072 with radioactivity of 174,859 Bq and 73,505 Bq, respectively) were excluded for statistical analysis. Radioactivity was significantly correlated with the weight and shortest diameter in both metastatic and non-metastatic LNs. When the data were fitted to first-order approximation equations, the slopes were higher in metastatic LNs than in non-metastatic LNs. A significant correlation between radioactivity and the longest diameter was observed only in non-metastatic LNs.

**Fig. 6:**
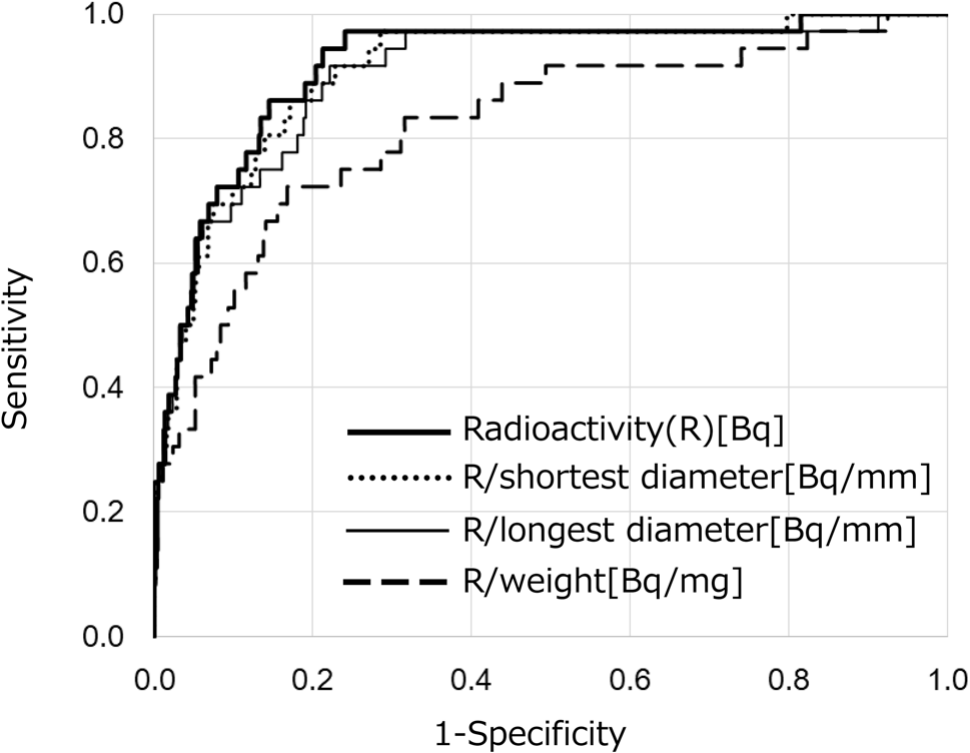
Receiver operating characteristic curves for radioactivity and adjusted measurements to discriminate between metastatic and non-metastatic lymph nodes. The values of area under the curve were 0.918 for radioactivity alone, 0.910 for radioactivity divided by the shortest diameter, 0.902 for radioactivity divided by the longest diameter, and 0.819 for radioactivity divided by the lymph node weight

To improve the diagnostic ability, we adjusted the radioactivity by dividing it by the weight, shortest diameter, or longest diameter of LNs. The consequent AUCs were 0.819, 0.910, and 0.902, respectively (Fig. 6). These values were lower than 0.918, which was obtained using LN radioactivity alone. The best cut-off value for radioactivity was 620 Bq without any adjustments.

To detect a radioactivity of 620 Bq assuming that the intraoperative measurement time was 30 s and the statistical error was limited to 10% in the measurement of radioactivity, we found that the efficiency of the detector needed to be 0.54% based on the following equations. The uncertainty of radioactivity decay is calculated by Eq. (1), where σ is the standard deviation of the measurement using the Poisson distribution and N is the total count recorded.

1 $${\upsigma } = \sqrt {{\text{N}},}$$

2 $$\% {\upsigma } = {{\sqrt {\text{N}} } \mathord{\left/ {\vphantom {{\sqrt {\text{N}} } {\text{N}}}} \right. \kern-\nulldelimiterspace} {\text{N}}} \times 100\left[ \% \right]$$

We supposed that %σ was 10% and N was 100.

3 $$620\left[ {{\text{Bq}}} \right] \times x \times 30\left[ {\text{s}} \right] = 100\left[ {{\text{counts}}} \right],$$

4 $$\% x = 0.0054 \times 100\left[ \% \right]$$

$x$ is the detector sensitivity, which is defined as the ratio of the number of counts per second recorded by the detector to the radioactivity.

The GEANT 4 simulation results showed a coincidence detection efficiency of 0.96%. Using the cut-off value of 620 Bq, we obtained the diagnostic capabilities of each node station (Table 1) [15]. False-negative results were observed in only two metastatic LNs. Among the total of 1035 non-metastatic LNs, 815 (78.7%) were below the cut-off value of 620 Bq at the assumed time of intraoperative measurement.

**Table 1:** Diagnostic capabilities of each node station using an estimated cut-off value of 620 Bq

Number	Name of node station (*JCEC*)	*n*	True		False	
			Positive	Negative	Positive	Negative
Cervical LNs						
101L	Cervical paraesophageal (L)	20	0	20	0	0
101R	Cervical paraesophageal (R)	32	4	19	9	0
104L	Supraclavicular (L)	103	0	82	21	0
104R	Supraclavicular (R)	75	2	65	8	0
Thoracic LNs						
105	Upper thoracic paraesophageal	18	0	13	5	0
106recL	Recurrent laryngeal nerve (L)	58	2	44	12	0
106recR	Recurrent laryngeal nerve (R)	58	2	44	11	1
106tbL	Tracheobronchia	21	0	17	4	0
107	Subcarinal	44	5	18	21	0
108	Middle thoracic paraesophageal	35	1	26	8	0
109L	Main bronchus (L)	49	3	31	15	0
109R	Main bronchus (R)	61	1	33	27	0
110	Lower thoracic paraesophageal	18	0	14	4	0
111	Supradiaphragmatic	22	0	21	1	0
112	Posterior Mediastinal	45	0	31	14	0
Abdominal LNs						
9	Along the celiac artery	33	0	30	3	0
8a	Along the common hepatic artery	21	0	13	8	0
7	Along the left gastric artery	41	1	34	6	0
3	Along the lesser curvature	205	11	161	32	1
2	Left cardiac	47	0	43	4	0
1	Right cardiac	45	3	38	4	0
4	Along the grater curvature	1	0	1	0	0
11	Along the splenic artery	19	1	16	2	0
12	In the hepatoduodenal ligament	2	0	1	1	0
	Total	1073	36	815	220	2

*JCEC* Japanese classification of esophageal cancer [15], *LN* lymph node, *L* left, *R* right

## Discussion

To achieve intraoperative LN diagnosis for patients with esophageal cancer, we proposed a new concept of a forceps-type coincidence detector composed of two small scintillation crystals that could detect pairs of annihilation radiation coincidentally. Based on the data including 1073 LNs from 20 patients with esophageal cancer, the most effective cut-off value of radioactivity to differentiate metastatic LNs from non-metastatic LNs was 620 Bq for each LN. To improve the diagnostic ability, we adjusted this value using the weight and the shortest or longest diameter of the LNs, since the size of the LNs could be estimated using the angle of the forceps tips. However, these attempts were not effective. Assuming that the intraoperative measurement time was 30 s and measurement statistical error was 10%, the detector sensitivity for radioactivity needs to be at least 0.54%. GEANT4 simulation result showed that one pair of BGO scintillation crystals with a size that allowed them to pass through a 12 mm diameter trocar achieved the detector sensitivity of 0.96%, which is higher than the required sensitivity. Actually, the detector needs other components, including photodetectors, electronic cables, container of the crystals as well as the scintillation crystals. A thin photodetector with a size of less than 4 × 4 × 2 mm^3^ and an electrical cable with a diameter of 1 mm are available; the frame supporting the crystals can be made of stainless steel with a thickness of 0.8 mm. Therefore, the size, including all the components needed for the detector, was estimated to be within 12 mm in diameter. Finally, we confirmed the feasibility of measuring the cut-off value of 620 Bq using the forceps-type coincidence detector intraoperatively within a 10% statistical error. We expect that the detector would provide LN diagnosis with a true-positive rate of 94.7% and true-negative rate of 78.7%.

The occurrence of postoperative pulmonary complications has been decreasing the overall survival rates [6, 16–19]. Although the less invasive surgical technique consisted of thoracoscopic and laparoscopic approach has been applied, pulmonary compilations have not been reduced [20, 21]. Postoperative pulmonary complications are caused by several factors such as infrahyoid muscle impairment due to cervical LN dissection [5] and reflux or swallowing dysfunction associated with recurrent laryngeal nerve impairment [22]. Therefore, the decision-making protocol regarding the extent of the dissected LN area is also needed. We proposed a new concept of the forceps-type coincidence detector to support the decision-making regarding intraoperative LN diagnosis. Among 1035 non-metastatic LNs, 815 (78.7%) showed radioactivity below the cut-off values. Particularly, 81.3% of the non-metastatic LNs in the cervical LN stations and 79.3% of the non-metastatic LNs in the recurrent laryngeal nerve LN stations showed radioactivity below the cut-off value. Therefore, precise intraoperative diagnosis of these non-metastatic LNs would have preserved them, avoiding the need for resection.

While considering the use of a forceps-type detector in the body cavity, it is necessary to consider the effect of background activity, such as physiological FDG uptake and primary tumors. Upon using the GEANT4 simulation, we estimated the random fraction of the forceps-type detector employed in thoracoscopic surgery. First, we assumed the FDG distribution of the whole body based on the package insert of FDG Injectable®, which is a commercially available FDG pharmaceutical, and the data of the Japanese standard organ weights [23]. Since the radioactivity in urine is drained during surgery, the radioactivity in the bladder is considered to be negligible. We thereafter set a clinical situation in which the recurrent laryngeal nerve LN with 620 Bq was measured using forceps-type PET in the case of a primary tumor of 10 mm diameter with a standardized uptake value of 10 in the middle thoracic esophagus. The random fraction was estimated to be 4.7% of all the coincident measurement counts. Therefore, we believe that the detector efficiency can be maintained when a forceps-type detector is used following insertion into the body cavity.

Radioguided surgery has been explored in the field of intraoperative identification of SLNs using low- to middle-energy gamma rays of ^99m^Tc-labeled pharmaceuticals wherein probe-type detectors are most commonly applied. Probe-type detectors are usually used along the outer surface of the body to detect sentinel LNs of breast cancer or malignant melanoma, which are located near the surface of the body, and ^99m^Tc-labeled pharmaceuticals are injected into the area close to the primary tumor. Therefore, there is nearly no background activity in the other organs. A combination of low-energy gamma ray and low background activity is suitable for probe-type detectors. However, since we are assuming the use of the detector in the body cavity during thoracoscopic or laparoscopic surgery to measure FDG, it is difficult for a probe-type detector to distinguish incoming gamma rays from the target from those from background radioactivity. Therefore, we propose a forceps-type detector in this study. In other studies, to overcome the difficulty of the probe-type detectors, three-dimensional (3D) images were generated by scanning from multiple directions with a mini gamma camera equipped with a position-tracking system in a previous study, in which the total measurement time was 45 s [24]. In addition, a small probe head was designed to be held by the forceps arm of a robot-assisted surgical system with a high flexibility of gamma-probe angles [25, 26]. This probe head succeeded in incorporating radio-guided surgery in minimally invasive surgery. For the detection of high-energy gamma rays such as the 511-keV rays, a probe-type detector requires a thicker mechanical collimator made of lead or tungsten, resulting in a detector diameter of 25 mm [27]. A larger size is problematic for use in laparoscopic surgery, since a typical trocar has an inner diameter of 12 mm. Recently, a coincidence imaging system composed of an external fixed detector and a small movable detector to detect pairs of 511-keV photons was proposed by Liyanaarachchi et al. [28]. They demonstrated the ^18^F source activity of 44 kBq by moving manually the probe for two min, but the detector sensitivity (0.29%) seemed insufficient for that uses with the distance between the detector and the subject. We showed that our proposed detector consisting of a pair of scintillation crystals would be able to maintain sufficient sensitivity to differentiate metastatic LNs despite a small crystal size. We believe that sufficient sensitivity of radiation detection is important to avoid false-negative results, which is a critical issue in cancer surgery.

An average of > 50 LNs per patient was resected during surgery for esophageal cancer. If each node is counted for 30 s, more than 25 min are needed for the evaluation of all the LNs during surgery. We believe that the assessment time using the forceps-type detector is acceptable because we can save time for the careful dissection of the LN regions if we judge them as non-metastatic LNs before dissection. In addition, LN regions that require measurement are areas associated with serious postoperative complications such as cervical LNs, recurrent laryngeal nerve LNs, and LNs around large blood vessels. Such LNs are estimated to account for approximately 40% of all LNs. Therefore, the measurement time will not have a significant effect on the overall surgery time.

The radioactivity values of metastatic and non-metastatic LNs overlapped in the range of 600 Bq–16,943 Bq. To improve the differentiation ability, we tried to adjust the radioactivity using the weight and the shortest or longest diameter of the LNs. However, these adjustments were not effective, since the radioactivity of non-metastatic LNs tended to increase with an increase in their size. Similar trend was observed for metastatic LNs. In addition, mediastinal LNs frequently showed higher FDG uptake in non-metastatic LNs with the highest false-positive rate of 53.8% for the subcarinal LN station, which was probably related to inflammation even in clinically stable patients [29]. This was one of the limitations of FDG as a biomarker for LN diagnosis.

We measured the exposure doses of the operating room staff, such as the surgeons, nurses, and anesthesiologists, during surgery in our previous study. The highest dose per procedure was 44 μSV for the surgeon. In similar studies on FDG-guided surgery using a probe-type detector, 2.5–8.6 μSV/h of absorbed radiation doses were reported when the surgery was performed 3 h following FDG (47.0 and 41.4 MBq) administration [30], and 164 μSV was reported when the surgery was performed 142 min following FDG injection (mean dose of 699.3 MBq) [31]. In an ex vivo FDG-PET study in which the surgery was performed 45 min following the administration of FDG (555–740 MBq), exposure doses for the surgical staff were reported to be 0.4–0.8 mSV per procedure [32]. The difference in the exposure dose is probably based on the injection dose and the time from administration to the start of surgery. The International Commission on Radiological Protection recommends that the exposure dose limit for healthcare workers is 50,000 μSV per year. Considering this dose, we believe that the procedure could be conducted within the occupational radiation exposure limits.

## Conclusion

To achieve intraoperative LN diagnosis using the proposed forceps-type coincidence detector, we determined that the best cut-off value for LN diagnosis was 620 Bq. The simulation results supported the compact detector design, which can achieve a sufficient coincidence efficiency. Based on data including 1073 resected LNs from patients with esophageal cancer, we concluded that approximately 80% of the prophylactic LN dissections would be avoided.
